# Cystathionine-γ-lyase overexpression modulates oxidized nicotinamide adenine dinucleotide biosynthesis and enhances neovascularization

**DOI:** 10.1016/j.jvssci.2022.11.003

**Published:** 2023-01-13

**Authors:** Kevin Kiesworo, Michael R. MacArthur, Peter Kip, Thomas Agius, Diane Macabrey, Martine Lambelet, Lauriane Hamard, C.-Keith Ozaki, James R. Mitchell, Sébastien Déglise, Sarah J. Mitchell, Florent Allagnat, Alban Longchamp

**Affiliations:** a Department of Vascular Surgery, Lausanne University Hospital, Lausanne, Switzerland; b Department of Biomedical Sciences, University of Lausanne, Lausanne, Switzerland; c Department of Health Sciences and Technology, ETH Zurich, Zurich, Switzerland; d Department of Surgery and Heart and Vascular Center, Brigham & Women's Hospital and Harvard Medical School, Boston, MA; e Department of Medicine, Lausanne University Hospital, Lausanne, Switzerland

**Keywords:** Hydrogen sulfide, CGL, Peripheral arterial disease, Endothelial cells

## Abstract

**Objective:**

Hydrogen sulfide is a proangiogenic gas produced primarily by the transsulfuration enzyme cystathionine-γ-lyase (CGL). CGL-dependent hydrogen sulfide production is required for neovascularization in models of peripheral arterial disease. However, the benefits of increasing endogenous CGL and its mechanism of action have not yet been elucidated.

**Methods:**

Male whole body CGL-overexpressing transgenic (CGL^Tg^) mice and wild-type (WT) littermates (C57BL/6J) were subjected to the hindlimb ischemia model (age, 10-12 weeks). Functional recovery was assessed via the treadmill exercise endurance test. Leg perfusion was measured by laser Doppler imaging and vascular endothelial-cadherin immunostaining. To examine the angiogenic potential, aortic ring sprouting assay and postnatal mouse retinal vasculature development studies were performed. Finally, comparative metabolomics analysis, oxidized/reduced nicotinamide adenine dinucleotide (NAD^+^/NADH) analysis, and quantitative real-time polymerase chain reaction were performed on CGL^WT^ and CGL^Tg^ gastrocnemius muscle.

**Results:**

The restoration of blood flow occurred more rapidly in CGL^Tg^ mice. Compared with the CGL^WT^ mice, the median ± standard deviation running distance and time were increased for the CGL^Tg^ mice after femoral artery ligation (159 ± 53 m vs 291 ± 74 m [*P* < .005] and 17 ± 4 minutes vs 27 ± 5 minutes [*P* < .05], respectively). Consistently, in the CGL^Tg^ ischemic gastrocnemius muscle, the capillary density was increased fourfold (0.05 ± 0.02 vs 0.20 ± 0.12; *P* < .005). Ex vivo, the endothelial cell (EC) sprouting length was increased in aorta isolated from CGL^Tg^ mice, especially when cultured in VEGFA (vascular endothelial growth factor A)-only media (63 ± 2 pixels vs 146 ± 52 pixels; *P* < .05). Metabolomics analysis demonstrated a higher level of niacinamide, a precursor of NAD^+^/NADH in the muscle of CGL^Tg^ mice (61.4 × 10^6^ ± 5.9 × 10^6^ vs 72.4 ± 7.7 × 10^6^ area under the curve; *P* < .05). Similarly, the NAD^+^ salvage pathway gene expression was increased in CGL^Tg^ gastrocnemius muscle. Finally, CGL overexpression or supplementation with the NAD^+^ precursor nicotinamide mononucleotide improved EC migration in vitro (wound closure: control, 35% ± 9%; CGL, 55% ± 11%; nicotinamide mononucleotide, 42% ± 13%; *P* < .05).

**Conclusions:**

Our results have demonstrated that CGL overexpression improves the neovascularization of skeletal muscle on hindlimb ischemia. These effects correlated with changes in the NAD pathway, which improved EC migration.

## Clinical Relevance

Peripheral arterial disease (PAD) currently affects >200 million people worldwide and is anticipated to increase with the aging population. In the present study, we outlined the beneficial effects of cystathionine-γ-lyase (CGL) overexpression in improving neovascularization and functional recovery in a murine PAD model. Taken together with other reported studies, our findings further highlight the untapped potential of targeting CGL for therapeutic interventions in patients with PAD.

**Article Highlights**

• **Type of Research:** Basic research in mice.
• **Key Findings:** CGL overexpression improves the neovascularization of skeletal muscle upon hindlimb ischemia. CGL overexpression increased endothelial cell migration and was associated with changes in the NAD pathway.
• **Take Home Message:** The results of our study indicate that hydrogen sulfide and CGL might facilitate recovery upon limb ischemia and during peripheral arterial disease.

Peripheral arterial disease (PAD) currently affects >200 million people worldwide and is anticipated to increase with the aging population.^1^ PAD can lead to severe complications such as chronic limb threatening ischemia and amputation and has been associated with high rates of cardiovascular events and death. Clinical management of PAD aims to improve patients’ functional capacity and maintain limb viability.^2^ During PAD, occlusion of the arteries can cause a series of compensatory events, such as arteriogenesis and angiogenesis, to restore perfusion to the ischemic tissue.^3^^,^^4^ One therapeutic strategy that has been explored for PAD has been to enhance the formation of a new capillary network through administration of growth factors and vasculogenic cells and subsequent activation, proliferation, and migration of endothelial cells (ECs). Although some success with angiogenic therapy has been reported in young and healthy animal models of acute artery ligation, these therapies have not yet demonstrated therapeutic efficacy when translated to patient cohorts.^3^^,^^5^^,^^6^

In mammals, hydrogen sulfide (H_2_S) is a ubiquitous redox modifying gasotransmitter that has numerous physiologic roles across various organ systems, including the cardiovascular system.^7^^,^^8^ H_2_S is produced through the transsulfuration pathway by the concerted effort of three enzymes: cystathionine-γ-lyase (CGL), cystathionine-β-synthase (CBS), and 3-mercaptopyruvate sulfurtransferase.^9^ In the cardiovascular system, CGL is thought to be the principal enzyme responsible for the production of H_2_S.^10^ *In vitro* and *in vivo* H_2_S promotes angiogenesis.^8^^,^^9^ In mice, whole body knockout of CGL impaired recovery in a murine model of PAD,^11^^,^^12^ and the administration of an H_2_S pro-drug has been shown to improve neovascularization in a porcine PAD model.^13^ In humans, endogenous H_2_S bioavailability is attenuated in the setting of chronic limb threatening ischemia and in patients with diabetes-related vascular inflammation.^14^ Moreover, our group recently demonstrated that circulating H_2_S levels will be lower in patients with atherosclerotic disease and that patients undergoing surgical revascularization with lower H_2_S production capacity will have higher rates of postoperative mortality.^15^

H_2_S-associated angiogenesis is thought to be driven by stimulation of the vascular endothelial growth factor A (VEGFA) pathway in ECs via activation of the VEGFR2 (vascular endothelial growth factor receptor 2) through sulfhydration.^16^ H_2_S further increases EC glucose uptake and adenosine triphosphate production, which allows for rapid energy generation, supporting migration during angiogenesis.^17^ A few studies have suggested an interplay between H_2_S and the redox couple oxidized/reduced nicotinamide adenine dinucleotide (NAD^+^/NADH). NAD^+^ is an essential coenzyme for cellular redox reactions, such as glycolysis and fatty acid oxidation, making it central to energy metabolism. Moreover, it also functions as a substrate for nonredox enzymes, such as sirtuins and poly(ADP-ribose) polymerases.^18^^,^^19^ The H_2_S donor sodium hydrosulfide can also activate the NAD^+^ dependent histone deacetylase sirtuin 1 (SIRT1) and augment the proangiogenic effects of the NAD^+^ precursor nicotinamide mononucleotide (NMN) in primary ECs^20^, ^21^, ^22^ and elderly mice.^20^ In muscle, supplementation with a NAD^+^ precursor, nicotinamide riboside, also accelerated regeneration in young and aged mice in a model of cardiotoxin-induced muscle damage.^23^ However, the intricacies of the interaction between H_2_S and NAD^+^ have not yet been elucidated.

To investigate the effect of H_2_S on neovascularization, we used a model of limb ischemia and transgenic mice overexpressing CGL (CGL^Tg^). We identified CGL as a potent proangiogenic trigger in vivo, which was associated with changes in the NAD^+^ pathway.

## Methods

The materials and reagents used are described in Supplementary Tables I-IV (online only).

### Mice

Male mice, aged 10 to 12 weeks, both wild type (WT) and CGL^Tg^ on a C57BL/6J genetic background, were used for all experiments. The CGL^Tg^ mice have been previously described.^24^ All mice were housed in standard housing conditions (22°C, 12-hour light/dark cycle), with ad libitum access to water and a regular diet (SAFE 150 SP-25 vegetal diet; SAFE Diets, Augy, France). All animal experiments conformed to the U.S. National Research Council’s *Guide for the Care* and *Use of Laboratory Animals*.^25^ The Lausanne University Hospital and the Cantonal Veterinary Office approved all animal care, surgery, and euthanasia procedures (SCAV-EXPANIM, authorization nos. 3258 and 3504).

### Hindlimb ischemia model

The hindlimb ischemia model was performed as previously described.^17^ In brief, the mice were anesthetized with isoflurane (2.5% with 2.5 L of oxygen), and the body temperature was maintained on a circulating heated pad. After a 1-cm groin incision, the neurovascular pedicle was visualized under a microscope (Z2 Zoom Stereo Microscope; LW Scientific, Lawrenceville, GA). The femoral nerve and vein were separated from the femoral artery. The femoral artery was ligated proximally and above both the proximal caudal femoral and superficial caudal epigastric arteries, allowing for electrocoagulation of the left common femoral artery and sparing the vein and nerve.^26^ Buprenorphine (Temgesic; 0.1 mg/kg; Reckitt Benckiser AG, Wallisellen-Zürich, Switzerland) was provided before surgery, with an analgesic administered postoperatively every 12 hours for 36 hours.

### Laser Doppler perfusion imaging

Laser Doppler perfusion imaging (LDPI) was performed as described previously.^17^ In brief, the mice were kept under isoflurane anesthesia, and the body temperature was maintained using a circulating heated pad. Once unconscious, we subjected the mouse hindlimbs to LDPI (Moor Instruments Ltd, Axminster, UK) with a low-intensity (2 mW) laser light beam (wavelength 632.8 nm). The hindlimb blood flow was recorded as a two-dimensional color-coded image, with a scan setting of 2 ms/pixel. Blood flow recovery was monitored at baseline and on days 0 (immediately after surgery), 1, 3, 5, 7, 10, and 14. The LDPI intensity of the ischemic foot was normalized to the corresponding contralateral foot and expressed as ratio between the ischemic and nonischemic limb.

### Treadmill test

At 14 days after hindlimb ischemia, the mice were acclimated to the treadmill (six-lane treadmill; Columbus Instruments, Columbus, OH) at 8 m/min for 5 minutes for 5 days before exercise testing. The next day, the mice were run until exhaustion at a 5° incline at 8 m/min for 10 minutes, followed by 10 m/min for 5 minutes, with a 2-m/min increase in speed every 5 minutes until exhaustion. Exhaustion was defined as follows: the mice had remained on the electric grid for 5 seconds, had remained on the electric grid for 25 seconds in total, had receive >25 shocks in 3 minutes, or had received >200 shocks in total.

### H_2_S production assay

Measurement of the H_2_S production capacity in tissues was performed as previously described.^17^^,^^27^ In brief, 80 μg of protein was incubated in 10 mM L-cysteine and 1 mM pyridoxal 5′-phosphate hydrate (Sigma-Aldrich, St Louis, MO), or 20 μL of plasma was incubated in 100 mM L-cysteine and 10 mM pyridoxal 5′-phosphate hydrate. This mixture was sealed under lead acetate paper and incubated until black lead sulfide precipitate was detected, but not saturated. The lead sulfide presence was quantified using Fiji software, version 1.53f51 (available at: http://fiji.sc/Fiji).

### NAD^+^/NADH quantification

The NAD^+^/NADH level was determined using the NAD/NADH assay kit (Abcam, Cambridge, UK) according to the manufacturer’s instructions. Pulverized tissue (20 mg) was dissolved in the assay buffer from the kit as suggested by the manufacturer and normalized to the protein content using the Pierce BCA protein assay kit (Thermo Fisher Scientific, Waltham, MA), with 250 μg of protein used for each reaction. The NAD^+^ and NADH concentrations are expressed as ng/mg protein.

### Metabolomics

Polar metabolite profiling was performed as described previously.^28^ In brief, 20 mg of mouse gastrocnemius tissue was homogenized on dry ice in 80% methanol and kept at −80°C overnight. The debris was pelleted and the methanol suspension dried under nitrogen. The resulting pellet was resuspended in water, and the metabolites were measured using targeted tandem mass spectrometry with polarity switching and selected reaction monitoring with an AB/SCIEX 6500 QTRAP mass spectrometer.

### Immunohistochemistry

Immunohistochemistry was performed on 10-μm frozen sections of gastrocnemius muscle. After 5 minutes of fixation in 4% paraformaldehyde and rinsing in phosphate-buffered saline (PBS), immunostaining was performed as previously described.^17^^,^^29^ The slides were incubated overnight with vascular endothelial-cadherin (VE-cad; 1:200; CD144; BD Pharmingen; BD Biosciences, Franklin Lakes, NJ) rat anti-mouse primary antibody, followed by AlexaFluor 488 anti-rat secondary antibody, and washed and mounted in DAPI (4′,6-diamidino-2-phenylindole)-containing Vectashield fluorescent mounting medium (Vector Laboratories, Burlingame, CA). The sections were then scanned using an Axioscan microscope (Carl Zeiss, Oberkochen, Germany). The VE-cad–positive area of whole muscle was quantified blindly using Fiji software, version 1.53f51 (available at: http://fiji.sc/Fiji). Quantifications are expressed as a percentage of the VE-cad–positive area to the total surface area of the gastrocnemius muscle.

### Immunostaining of whole retina mounts

The eyes from 5-day-old pups were harvested and fixed for 2 hours at 4°C in 1% to 4% paraformaldehyde, under gentle stirring. The retinas were isolated, stored in methanol at −20°C, and immunostained according to the reported protocols.^30^, ^31^, ^32^ The permeabilized retinas were incubated overnight at 4°C with biotinylated isolectin B4 (B-1205, diluted 1:500; Vector Laboratories). The retinas were imaged using a Zeiss LSM 780 GaAsp inverted laser scanning fluorescence microscope (Carl Zeiss). The vascular radial extension was quantified by dividing the area labeled by isolectin B4 by the total area of the retina using the angiogenesis analyzer plugin on Fiji (available at: http://fiji.sc/Fiji).

### Aortic ring sprouting assay

The aortic ring assay was performed as previously described.^33^ In brief, mouse thoracic aortas were isolated and cut into 1-mm-wide rings and embedded in Matrigel (Corning, Corning, NY) and incubated in EBM-2 (Lonza Inc, Basel, Switzerland) supplemented with VEGFA alone or full EC growth medium-2 (EGM-2) containing VEGFA, fibroblast growth factor, epidermal cell growth factor, and insulin-like growth factor-1 (Lonza Inc). The media were replaced every 2 days. For each sample, the length of eight sprouts at days 6 and 8, originating from the aorta, were quantified using the Fiji software (available at: http://fiji.sc/Fiji) from brightfield images taken at 2× magnification.

### Cell culture

Pooled human umbilical vein ECs (HUVECs; Lonza Inc) were maintained in EGM-2 (BulletKit; Lonza Inc) at 37°C, with 5% carbon dioxide and 5% oxygen, as previously described.^29^ Passages 1 to 8 were used for the described experiments.

### Cell migration assay

HUVECs were grown to confluence in a 12-well plate, and a scratch wound was created using a sterile P200 pipette tip, as previously described.^17^ Repopulation of the wound in the presence of mitomycin C was recorded using phase-contrast microscopy for 16 hours in a Nikon Ti2-E live-cell microscope (Nikon, Tokyo, Japan). The denuded area was measured at 0 hours and 10 hours after wound creation and quantified using ImageJ software, version 1.53f51 (available at: http://fiji.sc/Fiji). Data are expressed as a ratio of the healed area over the initial wound area.

### Cell proliferation assay

HUVECs were grown at 80% confluence (5 × 10^3^ cells per well) on glass coverslips in a 24-well plate and starved overnight in serum-free medium (EBM-2; Lonza Inc). Next, they were incubated for 24 hours in EGM-2 containing 10 μM bromodeoxyuridine. Immunostaining was performed on cells washed and fixed for 5 minutes in −20°C methanol, air-dried, rinsed in PBS, and permeabilized for 10 minutes in PBS supplemented with 2% bovine serum albumin and 0.1% Triton X-100. Bromodeoxyuridine-positive nuclei were automatically detected in ImageJ software, version 1.53f51 (available at: http://fiji.sc/Fiji) and normalized to the total number of DAPI-positive nuclei.^29^

### Western blotting

Gastrocnemius and soleus muscle samples were collected and flash-frozen in liquid nitrogen, ground to powder, and resuspended in sodium dodecyl sulfate (SDS) lysis buffer (62.5 mM TRIS; pH 6.8; 5% SDS; 10 mM EDTA [ethylenediaminetetraacetic acid]). The protein concentration was determined using the DC (detergent compatible) protein assay (Bio-Rad Laboratories, Reinach, Switzerland), and 10 to 20 μg of protein were loaded per well. The primary cells were washed once with ice-cold PBS and directly lysed with Laemmli buffer, as previously described.^29^^,^^34^ The lysates were resolved using SDS-PAGE (polyacrylamide gel electrophoresis) and transferred to a PVDF (polyvinylidene fluoride) membrane (Immobilon-P; Millipore AG, Altdorf, Switzerland). Immunoblot analyses were performed as previously described using the antibodies listed in Supplementary Table I (online only).^34^ Membranes were revealed by enhanced chemiluminescence (Immobilon; Millipore) using the Azure 280 device (Azure Biosystems, Dublin, CA) and analyzed using Fiji (ImageJ, version 1.53c). Protein abundance was normalized to total protein using the Pierce reversible protein stain kit for PVDF membranes (catalog no. 24,585; Thermo Fisher Scientific).

### Reverse transcription and quantitative polymerase chain reaction

Pulverized frozen gastrocnemius muscles were homogenized in Tripure isolation reagent (Roche, Basel, Switzerland), and total RNA was extracted as previously described.^17^ After RNA reverse transcription (Prime Script RT reagent; Takara Bio, Shagara, Japan), cDNA levels were measured using quantitative polymerase chain reaction (PCR) and the Fast SYBR Green Master Mix (reference no. 4,385,618; Applied Biosystems; Thermo Fisher Scientific) in a Quant Studio 5 Real-Time PCR System (Applied Biosystems, Thermo Fisher Scientific), using the primers detailed in Supplementary Table II (online only).

### Statistical analysis

The data are displayed as the mean ± standard error of the mean. Statistical significance was assessed in GraphPad Prism, version 9.1.0 (GraphPad, San Diego, CA) using the Student *t* test or one-way or two-way analysis of variance, unless otherwise specified. A *P* value of ≤ .05 was deemed statistically significant. Metabolomics data were analyzed using MetaboAnalyst software, version 5.0.^35^

## Results

First, we characterized CGL expression in the gastrocnemius muscles from WT (CGL^WT^) and CGL^Tg^ mice under baseline (nonischemic) conditions. As expected, CGL, but not CBS or 3-mercaptopyruvate sulfurtransferase, mRNA expression was increased in CGL^Tg^ muscle (Fig 1, A). Consistently, the CGL protein levels were 4.8-fold higher in the CGL^Tg^ gastrocnemius muscle (Fig 1, B). CGL immunostaining further showed that in the gastrocnemius muscle, CGL was mostly expressed by ECs and increased specifically in VE-cad–positive ECs on CGL overexpression (Fig 1, C). The number of VE-cad–positive ECs in the gastrocnemius muscle was similar in both CGL^WT^ and CGL^Tg^ mice (Fig 1, C and Supplementary Fig 1, online only). CGL overexpression correlated with an increase in H_2_S production in the whole muscle (1.5-fold; Fig 1, D) and serum (Fig 1, E). CGL and H_2_S production were also increased in the liver (Supplementary Fig 2, *A and D*, online only), kidney (Supplementary Fig 2, *B and E*, online only), and aorta (Supplementary Fig 2, *C and F*, online only) of CGL^Tg^ mice.

**Fig 1:**
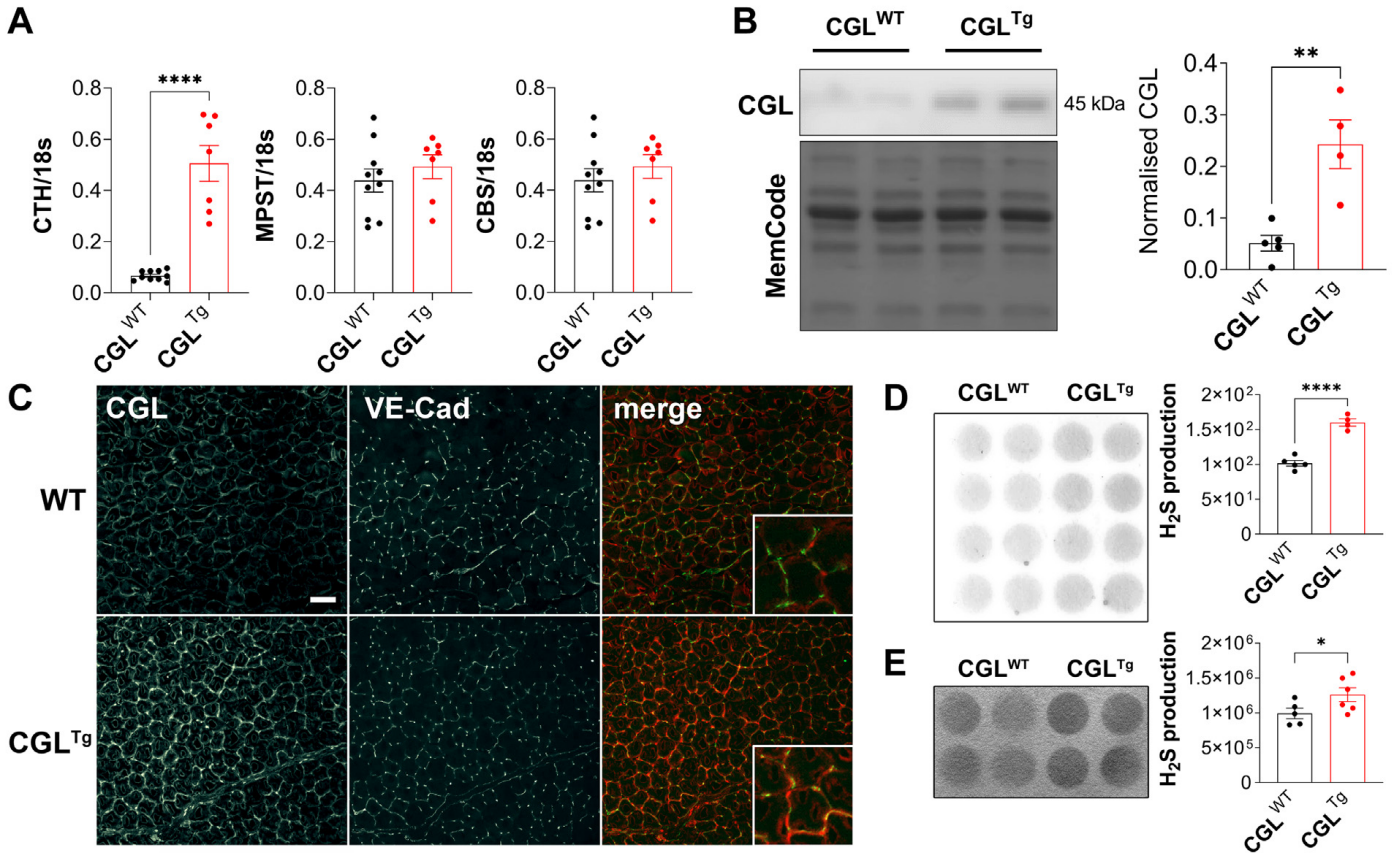
Hydrogen sulfide (H_2_S) production is enhanced in cystathionine-γ-lyase (CGL)-overexpressing (CGL^Tg^) gastrocnemius muscle. **A,** Quantitative real-time polymerase chain reaction (PCR) gene expression analysis of H_2_S-generating enzymes (CGL [CTH], 3-mercaptopyruvate sulfur transferase [MPST], and cystathionine-β-synthase [CBS]) in gastrocnemius muscle at baseline, normalized to 18S expression (n = 7-10 mice/group). **B,** CGL protein abundance in gastrocnemius muscle of CGL wild-type (CGL^WT^) and CGL^Tg^ mice at baseline by Western blot (Pierce reversible protein stain [Thermo Fisher Scientific] served as the loading control; n = 4-5 mice/group). **C,** Representative images of baseline gastrocnemius muscle sections (original magnification ×20) immunostained with CGL and vascular endothelial-cadherin (VE-cad) antibodies. Baseline CGL-mediated H_2_S production capacity in CGL^WT^ and CGL^Tg^ mice gastrocnemius muscle (**D**) and plasmatic H_2_S production (**E**) detected by lead acetate assay (n = 5-6 mice/group). Data presented as mean ± standard error of the mean ∗∗*P* ≤ .01 and ∗∗∗∗*P* ≤ .0001, Student's *t* test.

We previously reported that H_2_S/CGL triggers angiogenesis under amino acid restriction.^17^ We tested whether CGL overexpression alone was sufficient to promote neovascularization in the hindlimb ischemia model.^17^ Although the blood flow had been similarly interrupted in all mice immediately after ligation (day 0), the return of blood flow was accelerated in the CGL^Tg^ mice (Fig 2, A). The improvement in leg perfusion correlated with an increased running duration and distance run on a treadmill at 14 days after ligation (Fig 2, B). Immunostaining of gastrocnemius muscle sections at 21 days after ischemia induction indicated increased VE-cad–positive EC in the ischemic legs of the CGL^Tg^ mice (Fig 2, C) and reduced the muscle damage (Fig 2, D).

**Fig 2:**
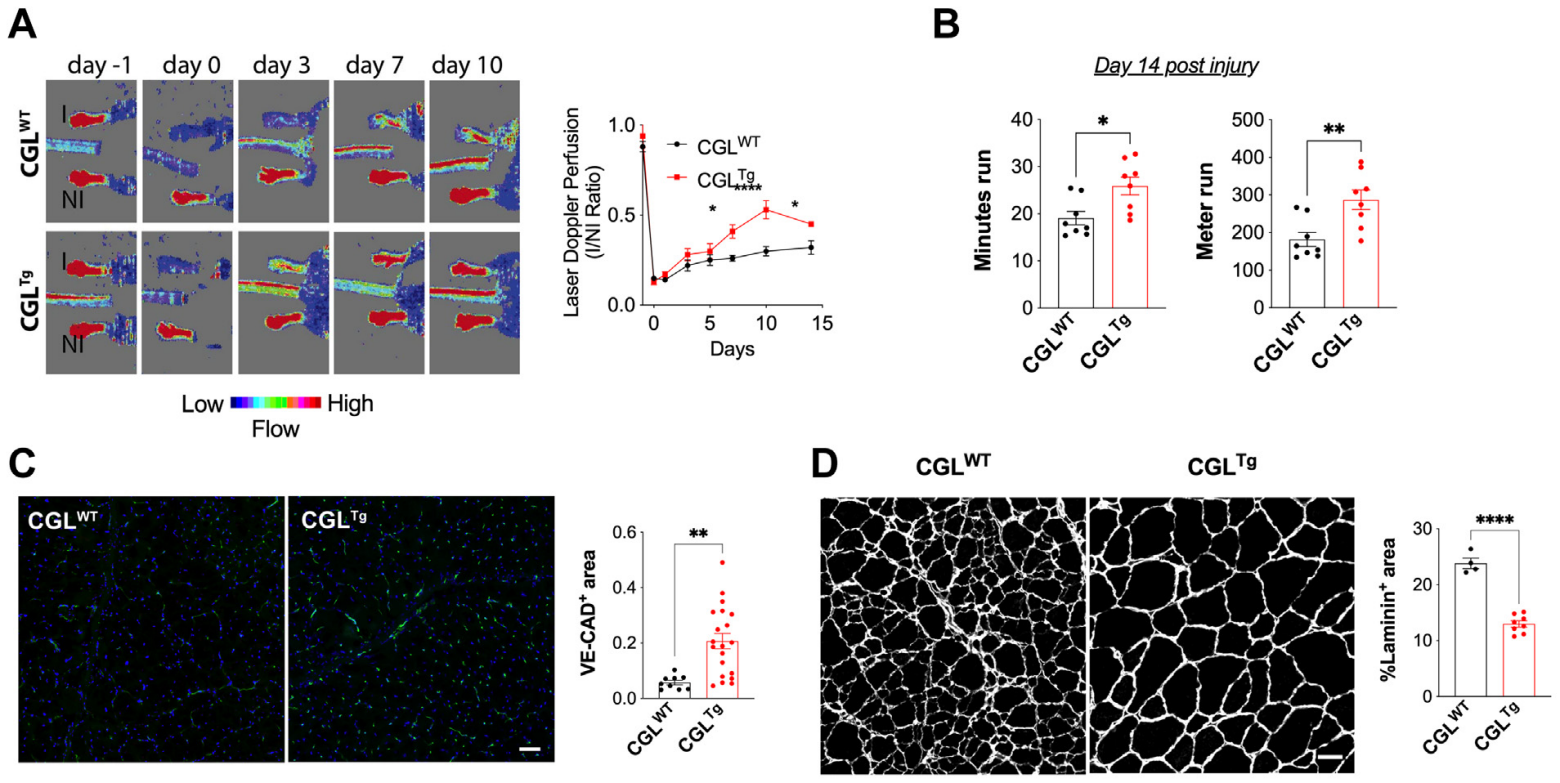
Cystathionine-γ-lyase (CGL)-overexpressing (CGL^Tg^) mice showed improved protection from hindlimb ischemia (*HLI*). **A,** Superficial perfusion of mouse hindlimb measured by laser Doppler perfusion imaging (LDPI). Results illustrated by representative LDPI images (*Left*) and ratio of LDPI perfusion quantification in ischemic and nonischemic limbs (*Right*; n = 8/group). **B,** Time (*Left*) and distance (*Right*) run on incremental speed test at day 14 after HLI (n = 8/group). **C,** Gastrocnemius muscle tissue perfusion 21 days after HLI shown in representative images and quantification (*Right*) of transverse sections stained with vascular endothelial-cadherin (VE-cad; n = 10-20/group). Scale bar = 50 μm. **D,** Representative transverse sections (*Left*) and quantification (*Right*) of laminin staining of gastrocnemius muscle at 21 days after HLI (n = 4-8/group). Scale bar = 50 μm. Data presented as mean ± standard error of the mean ∗*P* ≤ .05 and ∗∗*P* ≤ .01, Student's *t* test.

Next, we sought to better characterize the effects of CGL overexpression on sprouting angiogenesis using an aortic ring assay. In both complete EGM-2 and VEGFA-only media, vessel sprouting from aortic explants was significantly increased in the CGL^Tg^ mice compared with the CGL^WT^ mice, with a greater magnitude of increase with the VEGFA-only media (Fig 3, A and B). Developmental angiogenesis was not affected by CGL overexpression, as demonstrated by the pup retina vascular network at postnatal day 5 (Fig 3, C).

**Fig 3:**
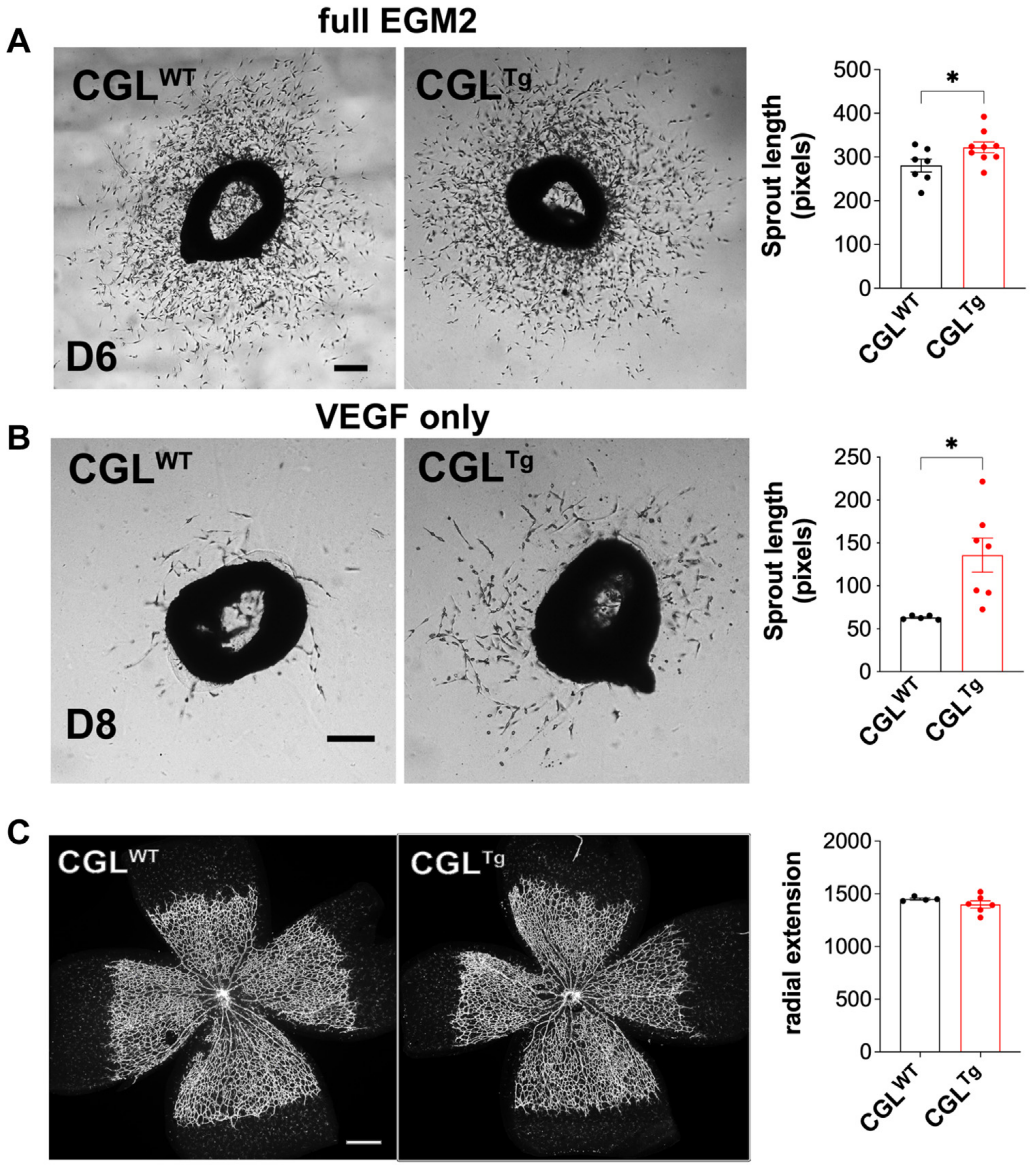
Vascular sprouting was accelerated in cystathionine-γ-lyase (CGL)-overexpressing (CGL^Tg^) mice. **A,** Average length of microvessel sprouting from aortic ring explants from wild-type CGL (CGL^WT^) and CGL^Tg^ mice incubated in full endothelial cell growth medium-2 (EGM-2; n = 7/group). Scale bar = 100 μm. **B,** Average length of microvessel sprouting from aortic ring explants from CGL^WT^ and CGL^Tg^ mice incubated in vascular endothelial growth factor A (VEGFA)-only EBM-2 (n = 7/group). Scale bar = 200 μm. **C,** Radial extension of developing mouse retina at postnatal day (*D*) 5, with representative images (*Left*) and quantification of radial extension (*Right*) of whole retina mounts stained with isolectin B4 (n = 4-5/group). Scale bar = 100 μm. Data presented as mean ± standard error of the mean ∗*P* ≤ .05, Student's *t* test.

**Fig 4:**
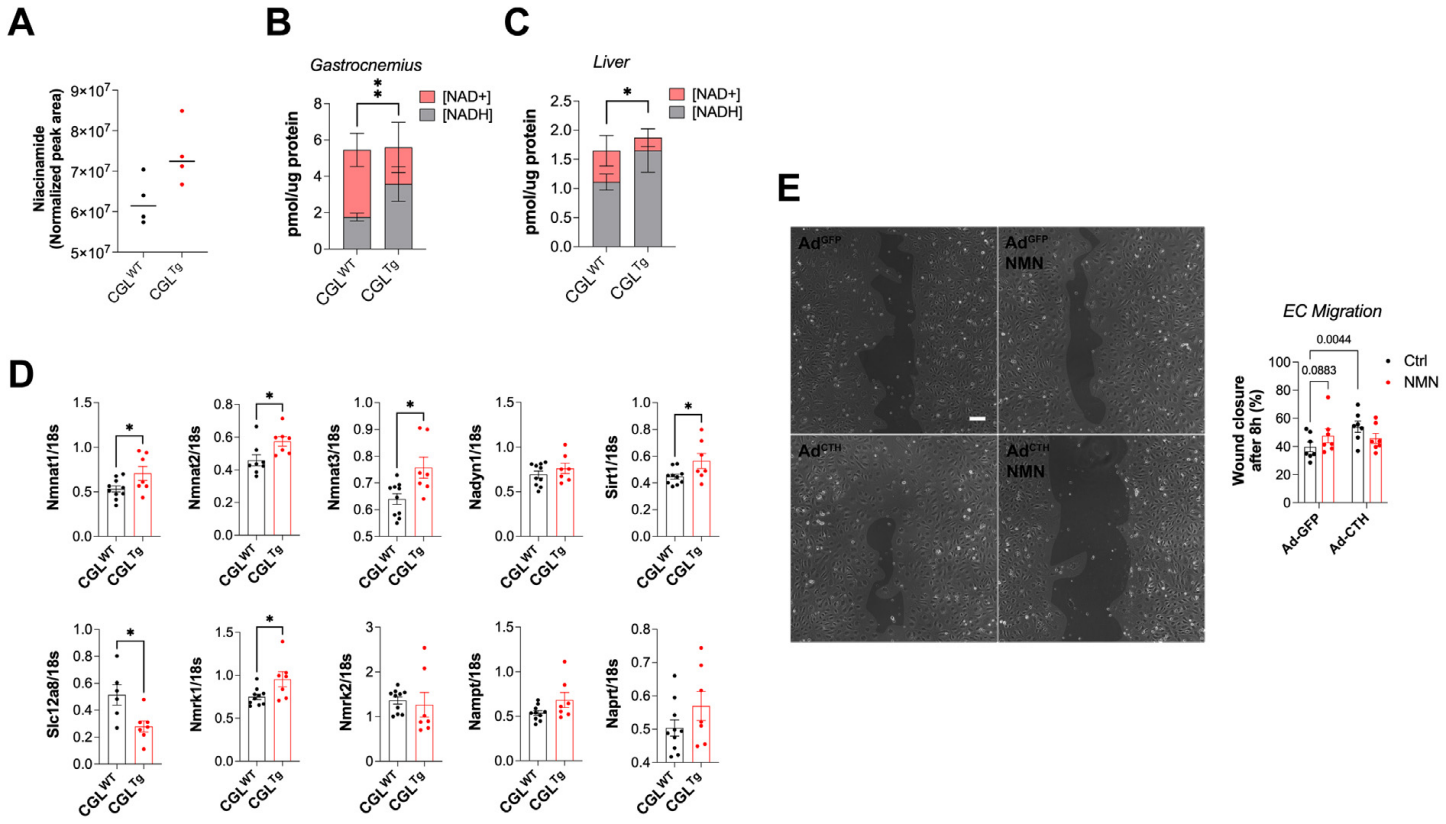
Beneficial effects seen in cystathionine-γ-lyase (CGL)-overexpressing (CGL^Tg^) mice was associated with changes in oxidized/reduced nicotinamide adenine dinucleotide (NAD^+^/NADH) pathways. **A,** Niacinamide level in CGL wild-type (CGL^WT^) and CGL^Tg^ gastrocnemius muscle (n = 4). *P* = .0569, Student's *t* test. **B,** Quantification of NAD^+^ and NADH concentration in gastrocnemius muscle (n = 5). Data presented as mean ± standard deviation. ∗∗*P* ≤ .01 (Student's *t* test), comparing NADH abundance between groups. **C,** Quantification of NAD^+^ and NADH concentration in liver (n = 5). Data presented as mean ± standard deviation. ∗*P* ≤ .05 (Student's *t* test), comparing NADH abundance between groups. **D,** Quantitative real-time polymerase chain reaction (PCR) gene expression analysis of enzymes associated with the NAD^+^/NADH pathway in gastrocnemius muscle. NAD-generating enzymes: Nmnat1, Nmnat2, Nmnat3, and Nadsyn1. NAD salvage pathway: Slc12a8, Nmrk1, Nmrk2, and Nampt. Preiss-Handler pathway: Naprt. NAD-consuming enzyme: SIRT1. All genes were normalized to 18S expression (n = 7-10/group). **E,** Endothelial cell (EC) migration across scratch with representative image (*Left*; original magnification, ×10) and quantification (*Right*) of human umbilical vein ECs (HUVECs) infected with green fluorescent protein (GFP)- or CGL-expressing adenovirus (*Ad*), with or without nicotinamide mononucleotide (NMN) supplementation, as indicated (n = 7/group). Scale bar = 100 μm. Data presented as mean ± standard error of the mean. ∗*P* ≤.05, Student's *t* test. *Ctrl,* Control.

To elucidate the mechanism of CGL-dependent angiogenesis, we performed targeted metabolomics on the baseline (nonischemic) CGL^WT^ and CGL^Tg^ mice. This revealed only three significantly differentially abundant metabolites; betaine (0.28 log2-fold change), kynurenic acid (−0.71 log2-fold change), and niacinamide (0.4 log2-fold change; Fig 4, A and Supplementary Table III, online only). The increase in niacinamide was of particular interest because of its role as a key precursor of NAD^+^/NADH.^20^ Consistently, the NADH level had increased twofold, and the NAD^+^ level had decreased equally in the gastrocnemius muscle (Fig 4, B) and liver (Fig 4, C) of the CGL^Tg^ mice. Quantitative PCR analyses of the genes associated with NAD^+^/NADH metabolism revealed increased expression of the NAD^+^ salvage pathway genes Nmnat1, Nmnat2, and Nmnat3 (Fig 4, D). Expression of SIRT1, a major NAD^+^ consuming enzyme, was also increased in the CGL^Tg^ mice (Fig 4, D).

Further in vitro experiments conducted in human ECs (HUVECs) indicated that adenoviral-mediated CGL overexpression or treatment with the NAD^+^ precursor NMN promoted HUVEC migration. However, the combination of CGL overexpression and NMN did not result in further improvement, suggesting a redundant effect on migration (Fig 4, E). CGL overexpression or NMN treatment had no effect on cell proliferation (Supplementary Fig 3, online only).

## Discussion

In the present study, we have demonstrated a beneficial role for CGL overexpression in a murine model of PAD. In addition, CGL overexpression modulated the NAD^+^/NADH biosynthesis pathway, which improved EC migration, angiogenesis, and recovery from hindlimb ischemia.

Our in vivo findings have built on previous work suggesting that H_2_S is beneficial for neovascularization of the ischemic hindlimb.^11^^,^^13^^,^^36^ CGL overexpression predominantly promoted VEGFA-dependent sprouting angiogenesis, building on our previous findings that amino acid restriction induces CGL overexpression and H_2_S production, which stimulates VEGFA-dependent EC migration and angiogenesis^17^ via persulfidation of VEGFR2.^16^^,^^37^

We observed that the NAD^+^/NADH precursor niacinamide was increased in the muscle of CGL-overexpressing mice and that the NAD^+^/NADH balance had shifted. Moreover, the expression of several key enzymes within the NAD^+^/NADH biosynthesis pathway changed in response to CGL expression. Prior in vitro studies of HUVECs demonstrated a transient increase in the NAD^+^ concentration during sodium hydrosulfide treatment.^20^ In the present study, the NAD^+^/NADH ratio was decreased in the gastrocnemius of the CGL-overexpressing mice. Our measurements of the steady state metabolite levels limited our interpretations, because differences could exist in the rate of NAD^+^ turnover or with differences in the shorter vs longer term effects of H_2_S augmentation. Further experiments using flux measurements with stable isotope-labeled substrate are required to determine the exact relationships and interplay between H_2_S and NAD^+^. An alternative explanation for the CGL-mediated reduced NAD^+^/NADH ratio could stem from an enhancement of glycolysis^17^^,^^38^ because glycolysis consumes NAD^+^ as a coenzyme.^19^ Thus, the increase in relative abundance of NADH in the CGL^Tg^ muscle might represent an increase in glycolysis-related NAD^+^ consumption. We previously demonstrated that CGL overexpression in ECs increased glycolysis and adenosine triphosphate production.^17^ Thus, it is likely that NADH accumulation is driven by NAD^+^ consumption, which is known to play a key role in neovascularization during limb ischemia.^20^ Despite our observations, the mechanistic understanding of the interplay between H_2_S and the NAD^+^/NADH system is still incomplete and warrants further investigation. Additionally, although the present study has demonstrated that CGL is sufficient in inducing the observed phenotype, it has not demonstrated that CGL is essential for NAD^+^ biosynthesis and neovascularization.^11^

In addition, SIRT1 gene expression was increased in CGL^Tg^ gastrocnemius. EC-specific SIRT1 knockout mice have impaired neovascularization and muscle endurance after hindlimb ischemia.^39^ Thus, the increased SIRT1 expression seen in CGL-overexpressing mice might support neovascularization. As a NAD^+^-consuming enzyme, SIRT1 increased expression could further reduce the NAD^+^ concentration in CGL^Tg^ mice. On a cellular level, we found that boosting H_2_S and NAD^+^/NADH production enhanced EC migration but not proliferation in vitro. This is consistent with prior studies, which highlighted substantial improvements in EC migration with H_2_S supplementation.^17^^,^^40^ In addition, boosting both H_2_S and NAD^+^/NADH pathways simultaneously did not have a synergistic effect on EC migration, suggesting that the promigratory effects of H_2_S and NAD^+^/NADH occur through the same mechanism. Endothelial tip cell migration drives sprouting angiogenesis,^41^ and we previously showed that H_2_S promotes VEGFA-dependent tip-cell migration.^17^ Thus, we propose that CGL overexpression drives VEGFA-induced tip-cell migration and sprouting angiogenesis to improve neovascularization of the ischemic muscle.

Although our study focused on changes within the NAD^+^/NADH pathways, our metabolomics analysis revealed two other significantly differentially abundant metabolites: betaine (0.28 log2-fold change) and kynurenic acid (−0.71 log2-fold change). Betaine is a part of the transulfuration pathway, allowing regeneration of methionine from homocysteine via the betaine homocysteine methyltransferase.^9^ Betaine accumulation is consistent with a shift toward the use of homocysteine to generate cysteine via CGL. The accumulation of kynurenic acid suggests a shift in the kynurenine pathway, outlining the conversion of tryptophan to NAD^+^. Most enzymes in this pathway are dependent on vitamin B_6_ pyridoxal 5′-phosphate, similar to the transulfuration pathway enzymes CGL and CBS. Accumulation of kynurenic acid could have resulted from reduced kynurenine pathway activity due to NAD+ accumulation or increased pyridoxal 5′-phosphate usage by CGL. Both the betaine and tryptophan/kynurenic acid pathways are important in the regulation of immune cells. Previous studies have shown that macrophages are polarized toward the pro-repair M2 phenotype in the presence of betaine.^42^ Moreover, the same phenomenon was observed in the context of indoleamine 2,3-dioxygenase overexpression, a key enzyme driving the catabolism of tryptophan to kynurenic acid.^43^ The M2 macrophage phenotype has been shown to be vital to neovascularization in the context of the hindlimb ischemia model^44^ and could, hence, posit a plausible mechanism of action to explain the improvements in neovascularization with CGL overexpression.

Although our findings have given us cause to be optimistic, we are aware of our study’s limitations. We endeavored to replicate PAD pathology with our hindlimb ischemia procedure. Although it might be the reference standard in replicating the pathology in vivo, it does not reflect the chronic nature of PAD development. We recently demonstrated that the H_2_S production capacity and plasma sulfide concentrations were reduced in patients with PAD.^15^ The chronicity of PAD might allow for the development of compensatory adaptations, absent in the hindlimb ischemia model. Furthermore, we have yet to fully characterize the potential off-target effects of CGL overexpression, which might induce adaptations independently of H_2_S. Thus, one could anticipate changes in the relative levels of sulfur-containing amino acids, which could have unforeseen effects on the ischemic adaptations. From our study, we only drew conclusions regarding the effects of the global increase in CGL; however, it remains to be seen whether the protection is due to CGL overexpression locally within ECs or due to distal systemic CGL overexpression. Because our long-term goal is to offer therapeutic angiogenesis, it will be important to identify the cell types and organ systems that drive the proangiogenic phenotype. In addition, EC migration assays were performed of HUVECs because they are readily available and able to migrate.^17^^,^^20^ However, HUVECs might not be representative of the actual in vivo conditions because they derive from immune-privileged fetal tissue. Thus, these data should be reproduced in arterial ECs (eg, human aortic ECs), which might be more closely related to what occurs in patients with PAD. Finally, only male mice were used in the present study; thus, the response to CGL overexpression might be different in female mice and deserves investigation in the future.

## Conclusions

In the present study, we have outlined the beneficial effects of CGL overexpression in improving neovascularization and functional recovery in a murine PAD model. Taken together with other reported studies, our study has further highlighted the untapped potential of targeting CGL for therapeutic interventions in patients with PAD.

## Author Contributions

Conception and design: KK, MM, PK, CO, JM, SD, SM, FA, AL

Analysis and interpretation: KK, MM, PK, TA, SM, FA, AL

Data collection: KK, MM, PK, TA, DM, ML, LH, FA, AL

Writing the article: KK, MM, TA, SM, FA, AL

Critical revision of the article: KK, PK, DM, ML, LH, CO, JM, SD, SM, AL

Final approval of the article: KK, MM, PK, TA, DM, ML, LH, CO, JM, SD, SM, FA, AL

Statistical analysis: KK, MM, FA, AL

Obtained funding: MM, JM, SD, FA, AL

Overall responsibility: AL

FA and AL contributed equally to this article and share co-senior authorship.

## Figures and Tables

**FigureS1.tiff:**
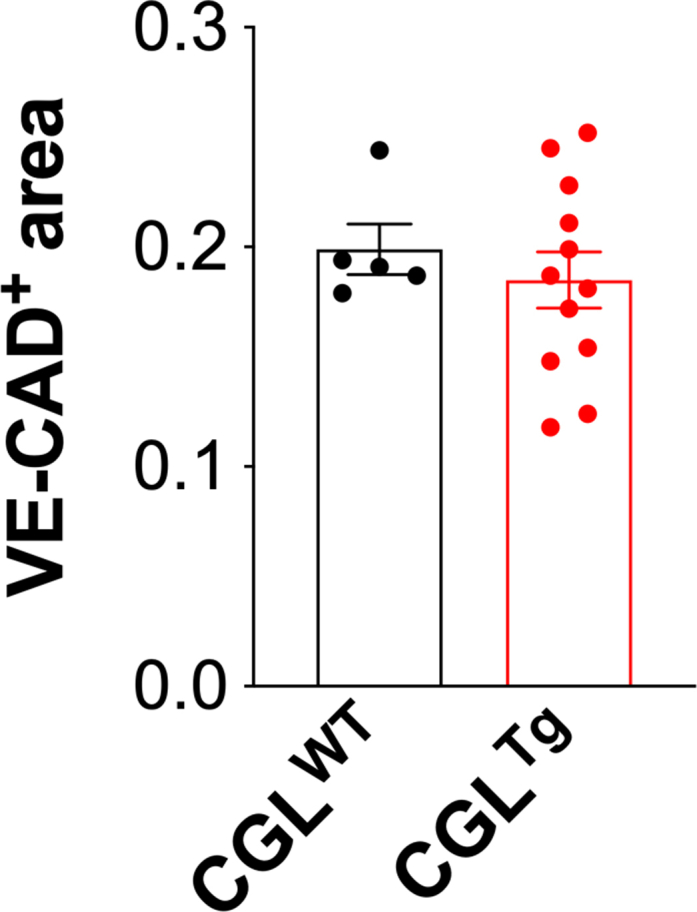


**FigureS2.tif:**
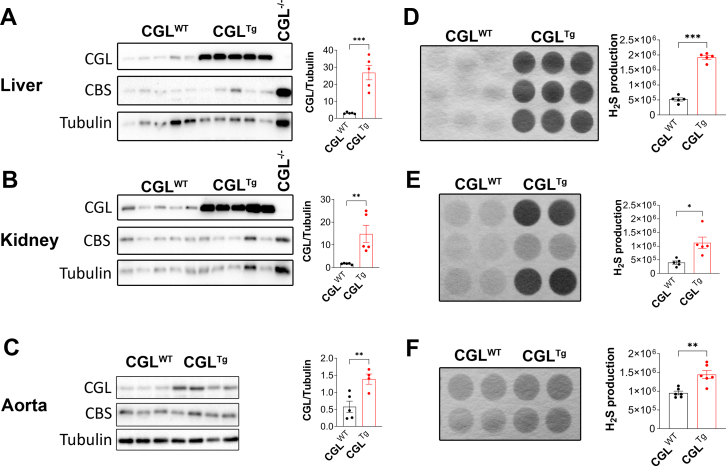


**FigureS3.tiff:**
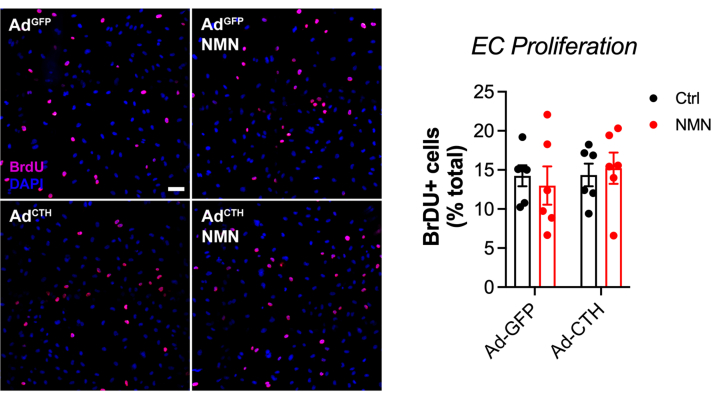


## Appendix (online only)

Supplementary Table S1.docx
