# Supplementary material for: Cystathionine-γ-lyase overexpression modulates oxidized nicotinamide adenine dinucleotide biosynthesis and enhances neovascularization
Source: JVS Vasc Sci. 2023 Jan 13;4:100095. doi: 10.1016/j.jvssci.2022.11.003 (PMC9958478; doi:10.1016/j.jvssci.2022.11.003)
Supplement: Supplementary Table S1.docx [file mmc1.docx]

**Supplementary Table S1: Antibodies**

| **Target antigen** | **Vendor** | **Catalog #** | **Working concentration** |
| --- | --- | --- | --- |
| Laminin | Sigma | L9393 | 1/200 |
| Ve-cadherin | Abcam | AB33168 | 1/100 |
| CGL | ProteinTech | 12217-1-AP | 1/1000 (WB)  1/100 (IHC) |
| Anti-Rabbit HRPO | Thermo Fisher Scientific | 31460A21109 | 1/20000 (WB)  1/250 (ICC) |
| Anti-mouse HRPO | Jackson ImmunoResearch Labs | 115-035-146 | 1/15000 (WB) |
| Anti-Rabbit HRPO | Thermo Fisher Scientific | 31460 | 1/20000 (WB) |
| BrdU | BD Biosciences | 555627 | 1/200 (ICC) |
| Goat anti-Rabbit IgG Secondary Antibody, Alexa Fluor 680 | Thermo Fisher Scientific | A21109 | 1/500 |
| Goat anti-Rabbit IgG Secondary Antibody, Alexa Fluor 405 | Thermo Fisher Scientific | A31556 | 1/500 |
| Goat anti-Rat IgG Secondary Antibody, Alexa Fluor 488 | Thermo Fisher Scientific | A11006 | 1/500 |
| Donkey anti-Rabbit IgG Secondary Antibody, Alexa Fluor 488 | Thermo Fisher Scientific | A21206 | 1/500 |

**Supplementary Table S2: DNA oligo primers**

| **Gene target** | **Sequence** |
| --- | --- |
| mouse CGL/CTH | F: TTGGATCGAAACACCCACAAA  R: AGCCGACTATTGAGGTCATCA |
| MPST | F: GGCCACCACTCTGTGTCATT  R: GGAGCTGATTGGCAGGTTCT |
| CBS | F: GGGACAAGGATCGAGTCTGGA  R: AGCACTGTGTGATAATGTGGG |
| Nmnat1 | F: ACAATGGCTGGGCCTTTAGA  R: CAGCGATGTTGTCTGCACCA |
| Nmnat2 | F: CCCATCATGACCGAGACCAC  R: CCCTGGCTCTCTCGAACATC |
| Nmnat3 | F: CATCAGCCTCTGCAGCACT  R: CCAAGCCGAACTTCTCCACT |
| Nadsyn1 | F: CTGGACACCACGCAGAATGG  R: TCATGGCACAGCCATCGTAG |
| Sirt1 | F: TACCTTGGAGCAGGTTGCAG  R: GCACCGAGGAACTACCTGAT |
| Slc12a8 | F: CTGGACCCAGAACATGGCTT  R: CCAGCCATGACTCCTGTAGC |
| Nmrk1 | F: AAACGCTCTTGAAGCTTGCTC  R: CTCCGTTTGTCACACCACCAA |
| Nmrk2 | F: AAACAGTGGGACGTGCTTGA  R: GCGTGCAAACTTGTGTGGAT |
| Nampt | F: TACTGTGGCGGGAATTGCTC  R: AGCCGTTATGGTACTGTGCTC |
| Naprt | F: TCAGCTACGCTAGCCTCGTA  R: GCCGCAAACCCATCTCTAGT |

**Supplementary Table S3: Metabolomics of baseline (non-ischemic) CGL^WT^ and CGL^Tg^ gastrocnemius muscle**

|  | FC | log2(FC) | raw.pval | log10P |
| --- | --- | --- | --- | --- |
| His-Cys-Lys-Ph-Trp-Trp | 0.519 | -0.947 | 0.0003 | 3.498 |
| betaine | 1.217 | 0.283 | 0.0135 | 1.871 |
| kynurenic acid | 0.610 | -0.714 | 0.0260 | 1.585 |
| Ala-Gln | 0.726 | -0.462 | 0.0291 | 1.536 |
| niacinamide | 1.316 | 0.396 | 0.0386 | 1.413 |
| spermine | 0.176 | -2.505 | 0.0576 | 1.239 |
| ornithine | 0.774 | -0.370 | 0.0583 | 1.234 |
| DMGV T2 | 0.763 | -0.389 | 0.0624 | 1.205 |
| Gly-Glu | 0.710 | -0.494 | 0.0857 | 1.067 |
| thyroxine | 1.514 | 0.599 | 0.0897 | 1.047 |
| arginosuccinate | 0.744 | -0.426 | 0.0897 | 1.047 |
| Dihydrouracil T1 | 1.419 | 0.505 | 0.1050 | 0.979 |
| Epinephrine | 1.534 | 0.617 | 0.1084 | 0.965 |
| GABA | 1.492 | 0.578 | 0.1084 | 0.965 |
| Tyr-Phe | 0.637 | -0.650 | 0.1161 | 0.935 |
| 3-OH Kyn | 0.517 | -0.952 | 0.1296 | 0.887 |
| methionine | 0.832 | -0.266 | 0.1305 | 0.885 |
| cysteamine | 0.828 | -0.272 | 0.1498 | 0.824 |
| 2'-deoxycytidine | 0.829 | -0.271 | 0.1516 | 0.819 |
| cytosine | 0.825 | -0.277 | 0.1522 | 0.817 |
| glutamine | 1.107 | 0.146 | 0.1557 | 0.808 |
| C181-carnitine | 1.497 | 0.582 | 0.1577 | 0.802 |
| PropionylCoA1 | 0.690 | -0.536 | 0.1699 | 0.770 |
| glutamate | 1.195 | 0.256 | 0.1742 | 0.759 |
| arginine | 1.321 | 0.401 | 0.1798 | 0.745 |
| Nicotinamide-N-Oxide | 0.727 | -0.459 | 0.1828 | 0.738 |
| aminoisobutyric acid | 1.436 | 0.522 | 0.1843 | 0.734 |
| trimethylamine-N-oxide | 0.792 | -0.336 | 0.1951 | 0.710 |
| carnitine | 1.143 | 0.192 | 0.1969 | 0.706 |
| N-Acetyl-L-Tyrosine | 0.654 | -0.614 | 0.1996 | 0.700 |
| tyrosine | 0.885 | -0.176 | 0.1998 | 0.699 |
| C18-carnitine | 1.394 | 0.479 | 0.2085 | 0.681 |
| creatine | 1.130 | 0.176 | 0.2133 | 0.671 |
| C16-carnitine | 1.602 | 0.680 | 0.2140 | 0.670 |
| lysine | 1.097 | 0.134 | 0.2156 | 0.666 |
| N-Acetyl-L-Alanine | 0.842 | -0.248 | 0.2228 | 0.652 |
| carnosine | 0.911 | -0.134 | 0.2351 | 0.629 |
| C182-carnitine | 1.539 | 0.622 | 0.2352 | 0.629 |
| glucose | 0.873 | -0.195 | 0.2411 | 0.618 |
| anserine | 0.913 | -0.131 | 0.2447 | 0.611 |
| thymidine | 0.718 | -0.478 | 0.2480 | 0.606 |
| N-carbomoyl-beta-alanine | 0.746 | -0.422 | 0.2570 | 0.590 |
| creatinine | 0.865 | -0.209 | 0.2700 | 0.569 |
| C4-methylmalonyl-carnitine | 1.117 | 0.160 | 0.2712 | 0.567 |
| 5-HIAA | 0.846 | -0.241 | 0.2892 | 0.539 |
| homocysteine | 0.737 | -0.440 | 0.2902 | 0.537 |
| cis/trans hydroxyproline | 1.169 | 0.225 | 0.2905 | 0.537 |
| alpha-glycerophosphocholine | 1.183 | 0.242 | 0.2949 | 0.530 |
| histamine | 0.847 | -0.239 | 0.2956 | 0.529 |
| spermidine | 1.385 | 0.470 | 0.2989 | 0.524 |
| N-Acetyl-L-Glutamine | 0.855 | -0.226 | 0.3042 | 0.517 |
| serine | 0.917 | -0.125 | 0.3184 | 0.497 |
| N-Acetyl-L-Methionine | 0.876 | -0.190 | 0.3210 | 0.494 |
| histidine | 0.875 | -0.192 | 0.3230 | 0.491 |
| Norepinephrine-updated | 0.905 | -0.143 | 0.3288 | 0.483 |
| valine | 0.868 | -0.205 | 0.3342 | 0.476 |
| cytidine | 0.913 | -0.131 | 0.3438 | 0.464 |
| Ala-Tyr | 1.227 | 0.295 | 0.3506 | 0.455 |
| Ala-Gly | 0.741 | -0.432 | 0.3521 | 0.453 |
| proline | 1.066 | 0.092 | 0.3539 | 0.451 |
| phenylalanine-d8 | 1.197 | 0.260 | 0.3600 | 0.444 |
| ADMA/SDMA | 1.148 | 0.200 | 0.3640 | 0.439 |
| allantoin | 0.880 | -0.184 | 0.3721 | 0.429 |
| C2-carnitine | 1.087 | 0.120 | 0.3742 | 0.427 |
| C244-carnitine | 3.022 | 1.595 | 0.3779 | 0.423 |
| Crotonyl-CoA2 | 1.132 | 0.178 | 0.3881 | 0.411 |
| N-Acetyl-L-Asparagine | 0.923 | -0.116 | 0.4102 | 0.387 |
| C3-malonyl-carnitine | 1.046 | 0.065 | 0.4109 | 0.386 |
| C4-butyryl-carnitines | 1.159 | 0.213 | 0.4127 | 0.384 |
| N-Acetyl-Lysine | 1.105 | 0.143 | 0.4311 | 0.365 |
| Ala-Leu | 1.264 | 0.338 | 0.4371 | 0.359 |
| C14-carnitine | 1.323 | 0.403 | 0.4413 | 0.355 |
| DMGV T1 | 0.868 | -0.205 | 0.4454 | 0.351 |
| leucine | 0.887 | -0.174 | 0.4702 | 0.328 |
| Cystathionine | 0.571 | -0.809 | 0.4879 | 0.312 |
| isoleucine | 0.885 | -0.176 | 0.4892 | 0.311 |
| NMMA | 1.134 | 0.181 | 0.4945 | 0.306 |
| Norepinephrine | 1.306 | 0.385 | 0.4964 | 0.304 |
| Glu-Gly | 0.829 | -0.271 | 0.5067 | 0.295 |
| xanthosine | 1.079 | 0.109 | 0.5085 | 0.294 |
| 5'-adenosylhomocysteine | 1.146 | 0.196 | 0.5086 | 0.294 |
| glycerol | 0.885 | -0.176 | 0.5227 | 0.282 |
| beta-alanine | 1.345 | 0.427 | 0.5393 | 0.268 |
| acetylcholine | 1.193 | 0.254 | 0.5406 | 0.267 |
| N1-Methylnicotinamide | 1.128 | 0.174 | 0.5425 | 0.266 |
| tryptophan | 0.900 | -0.152 | 0.5491 | 0.260 |
| thiamine | 0.932 | -0.102 | 0.5623 | 0.250 |
| xanthine | 1.144 | 0.194 | 0.5629 | 0.250 |
| Ala-Trp | 1.497 | 0.582 | 0.5814 | 0.236 |
| Cystine | 0.832 | -0.266 | 0.5928 | 0.227 |
| kynurenine | 1.095 | 0.131 | 0.6005 | 0.222 |
| phosphocholine | 1.100 | 0.138 | 0.6064 | 0.217 |
| C12-carnitine | 1.208 | 0.272 | 0.6289 | 0.201 |
| val-d8 | 1.033 | 0.047 | 0.6384 | 0.195 |
| serotonin | 0.913 | -0.131 | 0.6495 | 0.187 |
| Val-Tyr | 0.778 | -0.361 | 0.6512 | 0.186 |
| Cysteine | 0.932 | -0.101 | 0.6635 | 0.178 |
| 1-methylhistamine | 0.988 | -0.018 | 0.6695 | 0.174 |
| Malonyl-CoA1 | 1.062 | 0.087 | 0.6875 | 0.163 |
| N-Carbamoyl-BAIBA | 0.852 | -0.231 | 0.6919 | 0.160 |
| Dopamine-updated | 0.810 | -0.305 | 0.6931 | 0.159 |
| 3-hydroxyanthranilic acid | 1.057 | 0.079 | 0.7025 | 0.153 |
| xanthurenate | 0.925 | -0.113 | 0.7038 | 0.153 |
| Cys-Gly | 0.952 | -0.072 | 0.7225 | 0.141 |
| Val-Tyr-Val | 1.160 | 0.215 | 0.7282 | 0.138 |
| C5-glutaryl-carnitine | 0.960 | -0.059 | 0.7322 | 0.135 |
| cotinine | 1.445 | 0.531 | 0.7358 | 0.133 |
| citicholine | 0.958 | -0.062 | 0.7515 | 0.124 |
| C9-carnitine | 0.563 | -0.829 | 0.7635 | 0.117 |
| phenylalanine | 0.962 | -0.055 | 0.7663 | 0.116 |
| C10-carnitine | 1.101 | 0.138 | 0.7679 | 0.115 |
| N-Acetyl-L-Leucine | 0.981 | -0.028 | 0.7860 | 0.105 |
| alanine | 0.980 | -0.029 | 0.7863 | 0.104 |
| C6-carnitine | 1.066 | 0.092 | 0.7985 | 0.098 |
| asparagine | 1.027 | 0.038 | 0.8026 | 0.095 |
| Malonyl-CoA2 | 1.018 | 0.025 | 0.8053 | 0.094 |
| threonine | 1.018 | 0.025 | 0.8087 | 0.092 |
| glycine | 1.015 | 0.021 | 0.8113 | 0.091 |
| 2'-deoxyadenosine | 1.015 | 0.022 | 0.8132 | 0.090 |
| cAMP | 0.913 | -0.131 | 0.8234 | 0.084 |
| taurine | 1.015 | 0.022 | 0.8311 | 0.080 |
| citrulline | 1.017 | 0.024 | 0.8347 | 0.078 |
| N-Methyl-4-Pyridone-3-Carboxamide T1 | 1.021 | 0.030 | 0.8358 | 0.078 |
| C5-valeryl-carnitines | 0.941 | -0.087 | 0.8383 | 0.077 |
| N-Methyl-2-Pyridone-5-Carboxamide T1 | 1.020 | 0.028 | 0.8450 | 0.073 |
| anthranilic acid | 1.052 | 0.074 | 0.8489 | 0.071 |
| C3-carnitine | 1.117 | 0.160 | 0.8512 | 0.070 |
| N-Acetyl-L-Phenylalanine | 1.102 | 0.140 | 0.8552 | 0.068 |
| C7-carnitine | 0.805 | -0.313 | 0.8553 | 0.068 |
| Sucrose | 0.967 | -0.049 | 0.8592 | 0.066 |
| cystamine | 1.021 | 0.030 | 0.8624 | 0.064 |
| N-Methyl-4-Pyridone-3-Carboxamide T2 | 1.012 | 0.018 | 0.8624 | 0.064 |
| 5-hydroxytryptophan | 1.005 | 0.007 | 0.8627 | 0.064 |
| N-Methyl-2-Pyridone-5-Carboxamide T2 | 1.016 | 0.022 | 0.8701 | 0.060 |
| N-Acetyl-L-Ornithine | 1.002 | 0.003 | 0.8750 | 0.058 |
| dimethylglycine | 1.014 | 0.020 | 0.8859 | 0.053 |
| choline | 1.013 | 0.019 | 0.8924 | 0.049 |
| Gly-Asp | 0.980 | -0.029 | 0.8957 | 0.048 |
| 5-Adenosyl-Methionine SAMe | 0.968 | -0.046 | 0.9016 | 0.045 |
| Sarcosine | 0.990 | -0.014 | 0.9123 | 0.040 |
| Tyr-Ala | 1.016 | 0.023 | 0.9130 | 0.040 |
| cobalamin | 0.964 | -0.053 | 0.9208 | 0.036 |
| Gly-Gln | 1.006 | 0.009 | 0.9255 | 0.034 |
| Dopamine | 1.045 | 0.063 | 0.9315 | 0.031 |
| uridine | 1.129 | 0.175 | 0.9408 | 0.027 |
| N-Acetyl-L-Glutamic acid | 0.956 | -0.064 | 0.9549 | 0.020 |
| anandamide | 1.036 | 0.051 | 0.9554 | 0.020 |
| gamma-Glu-Cys | 1.016 | 0.023 | 0.9659 | 0.015 |
| C8-carnitine | 1.060 | 0.085 | 0.9784 | 0.009 |
| aspartate | 1.005 | 0.007 | 0.9828 | 0.008 |
| adenosine | 1.084 | 0.117 | 0.9902 | 0.004 |
| N-Acetyl-L-Isoleucine | 1.059 | 0.083 | 0.9969 | 0.001 |
